# Epigenetic Alterations and Inflammation as Emerging Use for the Advancement of Treatment in Non-Small Cell Lung Cancer

**DOI:** 10.3389/fimmu.2022.878740

**Published:** 2022-04-20

**Authors:** Shuo Yang, Yang Huang, Qi Zhao

**Affiliations:** ^1^ Guangzhou Municipal and Guangdong Provincial Key Laboratory of Molecular Target & Clinical Pharmacology, The NMPA and State Key Laboratory of Respiratory Disease, School of Pharmaceutical Sciences and The Fifth Affiliated Hospital, Guangzhou Medical University, Guangzhou, China; ^2^ Cancer Centre, Institute of Translational Medicine, Faculty of Health Sciences, University of Macau, Macau, Macau SAR, China; ^3^ MoE Frontiers Science Center for Precision Oncology, University of Macau, Macau, Macau SAR, China

**Keywords:** epigenetics, inflammation, NSCLC, immunotherapy, biomarker

## Abstract

Lung cancer remains one of the most common malignancies in the world. Nowadays, the most common lung cancer is non-small cell lung cancer (NSCLC), namely, adenocarcinoma, squamous cell carcinoma, and large cell lung carcinoma. Epigenetic alterations that refer to DNA methylation, histone modifications, and noncoding RNA expression, are now suggested to drive the genesis and development of NSCLC. Additionally, inflammation-related tumorigenesis also plays a vital role in cancer research and efforts have been attempted to reverse such condition. During the occurrence and development of inflammatory diseases, the immune component of inflammation may cause epigenetic changes, but it is not always certain whether the immune component itself or the stimulated host cells cause epigenetic changes. Moreover, the links between epigenetic alterations and cancer-related inflammation and their influences on the human cancer are not clear so far. Therefore, the connection between epigenetic drivers, inflammation, and NSCLC will be summarized. Investigation on such topic is most likely to shed light on the molecular and immunological mechanisms of epigenetic and inflammatory factors and promote the application of epigenetics in the innovative diagnostic and therapeutic strategies for NSCLC.

## Introduction

**Figure 1:**
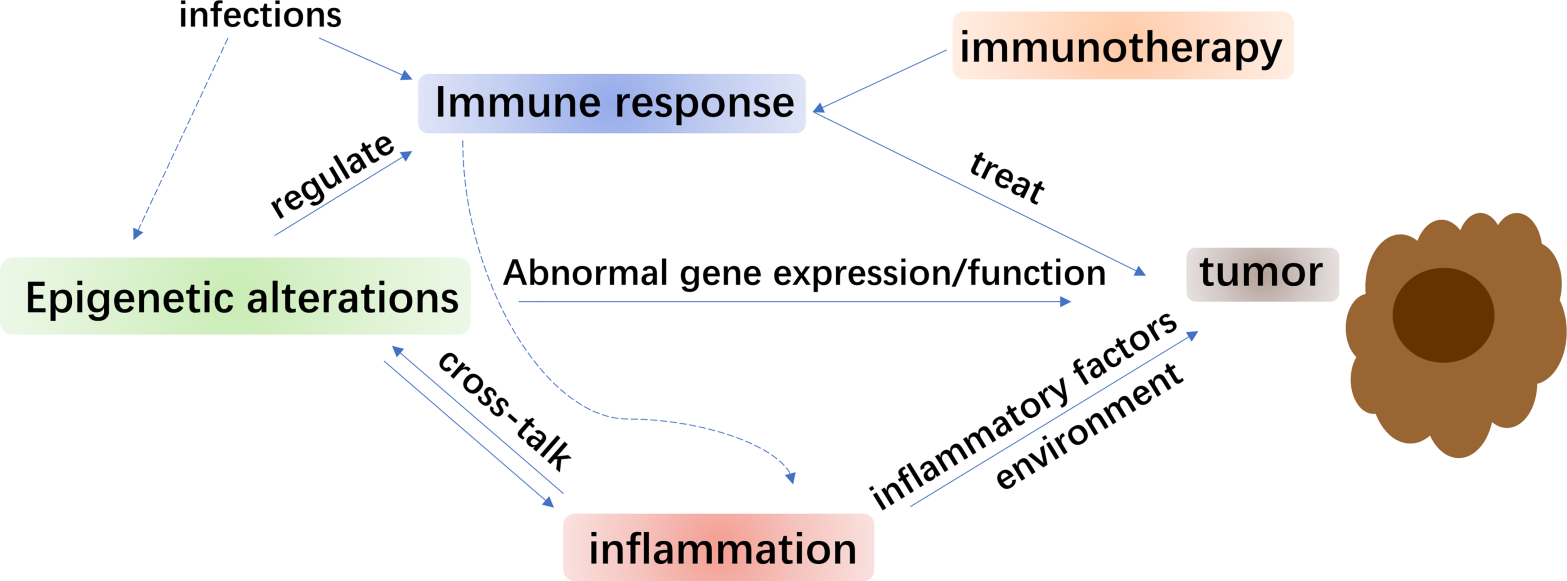
Schematic diagram of interaction.

Lung cancer is one of the most diagnosed cancers and the frequent cause of cancer-related deaths (1). It is broadly classified into two groups, namely, small cell lung cancer (SCLC) and non-small cell lung cancer (NSCLC) (2) (**Figure 1**). NSCLC is more common lung cancer with ∼85% of all lung cancer cases than SCLC. It is histologically categorized as squamous cell carcinoma (SCC), adenocarcinoma, and large-cell carcinoma (3). As is known, tyrosine kinase inhibitors (TKIs) and immunotherapy have provided considerable survival improvements for lots of patients of NSCLC. However, the levels of overall survival of NSCLC patients are still relatively low (4). Since such therapies could bring immune-associated adverse reactions and considerable financial requests (5, 6), an urgent demand emerges for vigorous predictive biomarkers and combination therapies.

Epigenetic regulating molecules are often dysregulated in cancer and offer a repertoire as promising therapeutic targets (7). Their contributions of epigenetic variations in the progression of different cancers could provide advancement of epigenetics-based therapies, such as targeting the DNA methyltransferases (DNMTs) or histone-modifying enzymes (8). Durable responses have been achieved in NSCLC patients who were originally treated with a DNA methyltransferase inhibitor (DNMTi) combined with interim histone deacetylase inhibitor (HDACi), followed by the immune checkpoint therapy (9). Combination epigenetic therapy regimes mostly use DNMTi in combination with HDACi for the latter can augment the re-expression of silenced genes regulated by abnormal gene promoter DNA methylation. The methylation patterns can offer conditions with molecular causes underlying the clinical advantage for immune checkpoint blockade (ICB) therapy (10, 11). Despite that first-line single agent ICB exhibited restricted activity in EGFR mutated NSCLC, the combination of immunotherapy and targeted agents has gained wide attention in both EGFR and ALK-positive NSCLC patients (12) Simultaneously, epigenetic marks led to the detection of potential cancer biomarkers for early screening, monitoring, and therapeutic methods of NSCLC (13).

Additionally, as a major environmental factor causing carcinogenesis and metastasis of NSCLC, cancer-associated inflammation and the inflammatory biomarker forecasting the clinical efficacy of TKIs and prognosis of NSCLC patients also need to be paid attention (14). Although only 20% of cancers are connected with chronic inflammation, innate immune cells and mediators are used to involve in most human malignancies (15, 16). The reason is the induction of inflammatory pathways in both malignant and pre-malignant cells triggered by oncogenic alterations. It was indicated that inflammation could result in cancer, while cancer could also lead to inflammation. Notably, inflammatory regulators mediate levels of enzymes that catalyze alterations of DNA methylation and histone structure, or change levels of non-coding RNAs (15).

In this study, we present the research of epigenetic and inflammatory drivers in prognosis and progression of NSCLC. The investigation on such topic could help improve the contributions of epigenetics in the innovative diagnostic and therapeutic strategies for NSCLC.

## Epigenetics in NSCLC

Epigenetic alterations, leading to abnormal gene expression without related changes in DNA sequence, can be inherited by cell division (17). Epigenetic regulation, namely, methylation, phosphorylation, acetylation, and ubiquitination are of importance in the modulation of gene expression (18). Three main epigenetic mechanisms involving DNA methylation, histone modification, and microRNA (miRNA) are mainly involved. Although it was proposed in 1983, the epigenetics of human cancer has not been shown in an essential position for human cancer genetics. But this subject became progressively noticeable since the developing elucidation of exact epigenetic mechanisms and their impacts on cancer (19). The beginning and development of lung cancer result from the interplay of permanent genetic and dynamic epigenetic alterations (20). It has been shown that several genes were detected as hypermethylated and downregulated in NSCLC cell lines under the action of inhibitors of DNA methylation and histone deacetylation, with gene expression profiling (21). Besides, mapping of DNA hypermethylation or hypomethylation discovered that expression of related target genes was suppressed or stimulated, respectively, in human NSCLC tissues (22). Epigenetic mechanisms of genes, especially target genes that influence the development of NSCLC might be used to investigate therapeutic possibilities for NSCLC. Epigenetic alterations referring to DNA methylation, histone modifications, and non-coding RNA expression driving the genesis and development of NSCLC will be discussed below.

### DNA Methylation

DNA methylation is crucial for normal progress and plays an essential role in many procedures, namely, transcriptional regulation, imprinting, and X-chromosome inactivation (23). It is regulated by three individual DNMTs: DNMT1, DNMT3A, and DNMT3B (24). As one of the best-studied epigenetic modifications, DNA methylation primarily occurs in CpG dinucleotides, where a methyl group is added to the carbon-5 position of cytosine. It is firmly determined as a dynamic balance in normal physiological conditions, as regulated by DNA methyltransferases and DNA demethylases, which shows a vital effect on the regulation of gene expression and the stabilization of heterochromatin structure (25). It has been revealed that gene-specific hypermethylation and genome-wide hypomethylation in the promoter of tumor suppressors are related to the formation of cancer (19, 26). DNA hypermethylation, a well-identified epigenetic change commonly at CpG islands (CGIs), is recognized to lead to the silence of gene expression and contributes to a reduction of cell growth (27–29). The mechanism underlying the silencing is stabilizing the structure of chromatin or interrupting the link between transcription factors and corresponding response elements of CpG sites (30). CGI hypermethylation leads to target gene silencing, involving genes with function referred to tumor suppressing, DNA repair and cell cycle control (20). Studies have shown that DNA methylation contributes to tumorigenesis and can be a potential tool for cancer detection and diagnosis over the past decade (31). It has been reported that, alterations of DNA methylation happen even prior to the emergence of atypical adenomatoid hyperplasia (AAH) during the progress of lung adenocarcinoma (LUAD) (32). Besides, several genes that downregulated in NSCLC by DNA methylation are relevant to epithelial–mesenchymal-transition (EMT), which is a conserved process related to decreased cell adhesion and increase of cell motility (33). Nowadays, methylation related genes have been generally investigated, incorporating RASSF1A (34), p16 (35), LRRC3B (36), EGLN2 (37), SETDB1 (38), and so forth. RASSF1A and p16 made great contribution to the cell cycle regulation while DNA methylation and expression of LRRC3B and EGLN2 could regulate hypoxia inducible factor 1A, which provided an effect on the early-stage NSCLC survival. SETDB1 was important for cell membrane recruitment, phosphorylation, and Akt activation underlying stimulation of growth factor. For NSCLC patients, gene methylation occurrence was reported to reach 96% (39). Among them, an upregulation of DNA methyltransferases expression was observed, namely, DNMT1, DNMT3A, and DNMT3B, which associated with silencing of tumor suppressor genes (TSGs) such as FHIT, RARβ, and CDKN2A (40, 41). Moreover, numbers of genes, such as CDH13, CDH1, DAPK, MGMT, p16, RASSF1A, etc., were demonstrated to hold intense promoter CGIs methylation in lung cancer, particularly for NSCLC (42, 43). In these probable TSGs, methylation of RARβ was in 40–43% of NSCLC, RASSF1A 30–40%, p16 25–41%, MGMT 16–27%, and DAPK 16–44% (43, 44). With the incidence and impacts of DNA methylation on meaningful target genes of NSCLC, it is vital to pay attention on such topic.

On the other hand, the incidence of hypomethylation was found relatively high by high-throughput analysis of genomic methylation among tumors (45, 46). The DNA hypomethylation that happened in CpG dinucleotides was the first discovered epigenetic aberration in cancer cells over almost four decades ago (47). It was also identified that the extent of DNA hypomethylation was positively related with progress of cancer (48), while global hypomethylation (49) and regional promoter CGIs hypomethylation (50–52) may trigger proto-oncogenes, loss gene imprinting, and revitalize the transposable elements. It was shown by high-resolution CpG methylation mapping that DNA hypomethylation appeared particularly at repetitive sequences in lung cancers (22), involving SINEs (short interspersed nuclear elements), LINEs (long interspersed nuclear elements), LTR (long terminal repeat) elements, heterochromatin repeats (e.g., satellite DNA), and segmental duplications in subtelomeric regions (20). For NSCLC, hypomethylation of LINE-1, a prognostic marker of cancer progress (53–55) has been identified as a cancer-specific epigenetic change, especially in squamous cell carcinoma (SqCC) (56) and often related to genomic instability (57). In addition, the LINE-1 hypomethylation level was revealed to be related with clinical progress and survival prognosis of NSCLC patients (58, 59). Based on the significant position of DNA methylation, it was noteworthy that ten-eleven translocation protein 1 (TET1) was found to present the ability to modify methylcytosine and intend to wipe off DNA methylation (60). Two other TET genes, namely, TET2 and TET3, were subsequently identified following sequence homology with TET1 (61). This family of proteins catalyzed the successive oxidation from 5-methylcytosine (5mC) to 5-hydroxymethylcytosine (5hmC), then to 5-formylcytosine (5-fC) and 5-carboxycytosine (5-caC) (62, 63). Intriguingly, the activity of TET enzymes became a pivotal tumor suppressor mechanism in cancer. It was shown that all three TET genes were mutated with reduced expression, and the proteins decreased activity among different kinds of cancer (64). Once balanced methylation pattern mediated by proteins like TETs and cofactors was interrupted, tumor suppressor genes can be repressed or oncogenes were activated, leading to various types of cancer. Recently, it was reported that hydroxymethylation levels were changed in cancer with several gene mutations that affected hydroxymethylation (23, 65–67). As mentioned, hydroxymethylation can be regulated by TET proteins and partly mediated by miRNAs. It is negatively related to cell proliferation while levels of 5hmC are reduced in growing tissues and cancer (68, 69).

Comprehensive understanding of the dynamics of DNA modifications is useful for a better demonstration of epigenetic regulation in solid tumors including NSCLC. Erasing DNA methylation can be achieved by DNA replication during cell division, or through oxidation of the methyl group by enzymes of the TET family (TET1, 2, 3). The above cases indicate that DNA methylation shows a close connection with cancer and help to find candidate treating targets for NSCLC.

### Histone Modification

Chromatin is formed by genomic DNA along with octamers of two copies of histone proteins H2A, H2B, H3, and H4. The N-termini of H3 and H4 histones involves several kinds of modifications, namely, acetylation, methylation, phosphorylation, ubiquitination and adenosine diphosphate (ADP) ribosylation (70–72). Histone acetylation loosens the bond between DNA and histone proteins thus the unpacked DNA with high affinity for RNA polymerase II and transcription factor promotes the transcriptional activation. The dynamic equilibrium of histone acetylation is maintained by histone acetyltransferases (HATs) and histone deacetylases (HDACs) (73). It was found that HDACs overexpress in several cancers and appeared to have potential as therapeutic targets (18). HDAC overexpression can lead to TSG silencing or altered transcription by influencing genes encoding HAT enzymes or binding elements of HAT and HDAC enzymes, which are associated with carcinogenesis (13). Similarly, histone methyltransferases (HMTs) and histone demethylases (KDMs) regulate the histone methylation dynamically (74). Enhanced transcription has been reported to be associated with high degrees of acetylation and trimethylation among histone 3 lysine 4 (H3K4me3), lysine 79 (H3K79me3), and lysine 36 (H3K36me3) (75). Histone modification commonly regulates gene expression along with DNA methylation in transcription levels (76).

Histone modifications play a critical role in human NSCLC. Global modification status of histone H3 and H4 in 408 NSCLC tissues was evaluated immunohistochemically, with results indicating that patterns of global histone H3 and H4 modification associated with tumor recrudesce and survival of NSCLC. Besides, for the squamous cell or large-cell carcinoma patients, higher degrees of H3K4 dimethylation represent potential better survival, while for the adenocarcinoma patients, lower degrees of H3K9 acetylation represent potential better survival (77). Li et al. (78) used the TCGA and cBioportal databases to evaluate the expression profile of methyltransferases and demethylases in NSCLC and found that higher expression of H3K4 histone demethylases (KDM1A, KDM5A, KDM5B and KDM5D) was associated with poor overall survival while patients with low expression of H3K4 histone methyltransferases SMYD3 also suffered from worse prognosis. In addition, high expression of KDM6A and enhancer of zeste homolog 2 (EZH2), mediators of H3K27 histone methylation provided poor overall survival prediction. Since methylation of lysine 27 of histone H3 (H3K27me3) has been demonstrated as a key regulator of transcriptional gene inhibition that maintains the normal biological activities (79–81), with whose disorder can result in various diseases and tumors (81, 82), Leng et al. (83) examined KDM6A, a member of the mixed-lineage-leukemia (MLL2) H3K4 methyltransferase complex, that catalyzes H3K27me2/3 with its JumonjiC (JmjC) domain (84, 85) and found that expression of KDM6A protein was higher in NSCLC tissues than that in the corresponding paracancer tissues while high KDM6A expression was positively related to the poor prognosis of patients. Mechanically, KDM6A colocalized and collaborated with KMT2B to regulate the transcriptional network by mediating the cancer pathway containing Wnt pathway as the major element. It is inferred that histone modifications and related molecules present in difference in NSCLC and normal samples that is valuable for relative studies.

Enhancers are cis-regulatory elements that could modulate type-specific gene expression (86, 87). Enhancers showed certain histone modifications of H3K4me1 and H3K27ac (88, 89). Loss of H3K4me1 by the depletion or mutation of histone methyltransferases MLL3 (KMT2C) and MLL4 (KMT2D) reversely affected H3K27ac at enhancers and led to transcriptional inhibition of target genes among mammalian cells (90–92). Large regulatory elements, called super-enhancers (SEs), were essential to the maintenance of cancer cell identity, and promoted oncogenic transcription to which cancer cells greatly relied on. Yuan and colleagues examined the H3K27ac landscape in two Chinese patient-derived LUAD cell lines and successfully discovered SE-associated gene RAI14 as a novel biomarker (93). Differentially methylated promoters and enhancers between PD-1 inhibitors responders and non-responders were finally detected, which may promote the development of biomarkers and therapeutic strategies for current anti-PD-1 immunotherapy in NSCLC.

Until now, histone modifying agents for NSCLC have been developed but its clinical application is still in early stages, which needs more large-scale clinical trials. Thus, further research on histone modifications and other factors in NSCLC from the perspective indicating biomarkers or treating targets would be meaningful for NSCLC patients.

### RNA-Based/Non-Coding RNA (ncRNAs) Alterations

There are numerous unique non-coding RNA (ncRNA) sequences spread over cells. Research has changed our previous opinion of ncRNAs from useless transcriptional products to functional mediators that regulate cellular procedures containing transcription, post-transcriptional alterations, chromatin remodeling and signal transduction (94). Developments of sequencing technologies have brought findings of various ncRNA types, such as microRNAs (miRNAs), transcribed ultra­conserved regions (95), circular RNAs (circRNAs), and long ncRNAs (lncRNAs) that mostly lack conservation among different species (96). The miRNAs commonly consisted of 20–22 nucleotides that can bind and act on mRNA, which regulate gene expression at post-transcription levels by leading to degradation of mRNA or inhibition of protein translation (97). It has been reported that miRNAs have a number of targets among various cancers and affect the cancerous development by upregulating oncogenes or downregulating genes of tumor suppressor (98). For example, miR-196b-5p was reported to enhance cell migration, proliferation, and tumor growth by affecting the tumor suppressors, TSPAN12, and GATA6 while increased miR-196b-5p expression in NSCLC was partly monitored by hypomethylation of its promoter section (99). Moreover, miR-142-3p was demonstrated to regulate starvation-induced autophagy of NSCLC cells by directly suppressing HMGB1 and consequently triggering the PI3K/Akt/mTOR pathway. In addition, overexpression of miR-142-3p can hinder antitumor drug-caused autophagy and improved chemo-sensitivity of NSCLC both *in vitro* and *in vivo* (100).

On the other hand, as one kind of non-coding RNA, lncRNAs can regulate gene expression on several stages, namely, the epigenetic, transcription, and post-transcription levels (101, 102). Linc00673 was validated as an oncogenic lncRNA in NSCLC and its expression was related to the poor prognosis of NSCLC while miR-150-5p can target linc00673 and influence its silencing induced proliferation, migration, invasion, and EMT suppressing effect in NSCLC (103). LINC01123 was found to overexpress in 92 paired NSCLC tissues and positively relate to poor outcomes of patients. Further analysis suggested that LINC01123 can increase NSCLC cell proliferation together with aerobic glycolysis by directly interacting with miR-199a-5p afterwards target and upregulating c-Myc (104). LncRNA gastric cancer−associated transcript 1 (GACAT1) was upregulated in NSCLC tissues and associated with survival. Mechanically, GACAT1 can negatively regulate miR−422a with YY1 transcription factor as a downstream target (105). Moreover, Circular RNA hsa_circ_0008305 (circPTK2) and transcriptional intermediary factor 1 γ (TIF1γ) were verified considerably downregulated in NSCLC cells undergoing EMT and tumor metastasis induced by TGF-β, indicating circPTK2 as a prospective therapeutic target for advanced NSCLC (106).

Taken together, the above research proposes that epigenetic alterations of certain functional genes are strongly involved in NSCLC. Several epigenetic alteration related genes may serve as promising predictors of prognosis or treatment targets for NSCLC.

## Inflammation in NSCLC and Immune Component of Inflammation

The existence of leukocytes within tumors, discovered by Rudolf Virchow in the 19th century, presented the first potential connection between inflammation and cancer (107). However, it was until the past decade that it became clear that there were potent interactions between inflammation and tumorigenesis (108). Inflammation is recognized as an essential innate immune response to disturbed tissue homeostasis. Chronic inflammatory activities play pivotal roles in all stages of tumor progress and affect the therapeutic outcomes (109). Inflammation refers to crosstalk across different immune cells, inflammatory cells, cytokines, chemokines, and proinflammatory regulators. It also makes contributions at several levels of tumor progress, involving initiation, promotion, invasion, malignant conversion and metastasis (110). For some kinds of cancer, inflammatory circumstances appear before a malignant conversion happens. In contrast, for other cancers, an oncogenic conversion generates a microenvironment of inflammation that fosters the progress of tumors (111).

In addition to that, chronic obstructive pulmonary disease (COPD) is a syndrome identified as an aberrant local and systemic inflammatory reaction, which is significantly related with lung cancer (112, 113). Data showed that impairment of lung function, the physiological character of COPD, was related to heightened systemic inflammation markers. Particularly, forced expiratory volume in one second (FEV1) was reported to have a reverse association with C-reactive protein, an effective marker of inflammation (114). Besides, the inflammatory enzyme cyclooxygenase-2 (COX-2) was found to overexpress in substantial malignancies (115) and its expression in NSCLC was related to angiogenesis (116, 117), metastasis (118, 119), and apoptosis resistance (120), the tumorigenic effects of which were partly regulated by the metabolite of COX-2, prostaglandin E2 (PGE2) that plentiful within the lung tumor microenvironment (TME). It was identified that overexpression and following mutation of the p53 gene that associated with inflammation/fibrosis-related oxidative DNA damage and restoration may promote the formation of a pro-tumor environment in patients suffering from idiopathic pulmonary fibrosis (121). Moreover, macrophages are omnipresent immune cells that charge many essential physiological and host protective activities, such as phagocytosis of pathogen, inflammation regulation, and tissue mending. Tumor associated macrophages (TAMs) that encompass a major portion of the leukocyte infiltrate characteristic of tumor can interplay with cancer cells and generate plenty of cytokines and growth factors that affect the regional microenvironment (122). Recently, more and more evidence appeared to support the opinion that macrophages influence tumor development by promoting proliferation, migration, and metastasis of cancer cell, facilitating angiogenesis, and inhibiting host antitumor immunity. However, there is substantial discussion concerning the prognostic relativity of TAMs in NSCLC (122, 123). Although some research demonstrated that expanded an number of TAMs present a benefit of survival, others considered it to indicate a poor consequence. Also, inflammation is regarded as a key promotor for the cancer development and progression (124). In addition, platelets that take part in an inflammatory process and make thrombocytosis become a common symptom in solid tumors (125). Therefore, neutrophil-to-lymphocyte ratio (NLR) and platelet-to-lymphocyte ratio (PLR) are well-known prognostic markers that are related to worse overall survival in several kinds of tumor containing NSCLC in the pre-immunotherapy period (126–129).

The adaptive immune response can be divided into two vital and complementary parts: humoral immunity and cell-mediated immunity. The CD4 T-helper (TH) lymphocyte is a crucial component for the two patterns above and can coordinate differently. However, it could overlap cytokine systems that affect other effector cells and in turn charge the form of the inflammatory reaction. CD4^+^ T helper 17 (Th17) cells, as a novel subset of the CD4^+^ helper T cells, are defined as production of interleukin (IL) 17A (IL-17A) and IL-17F (130). Th17 cells play crucial roles in inflammation and the progress of a tumor. The effects of Th17 cells in COPD-type inflammation-related lung cancer were confirmed by animal models (131) and the amounts of IL-17^+^ cells in NSCLC patients were observed to be positive-related to smoking conditions with poor survival rates (132). On the other hand, toll-like receptors (TLRs) were pivotal receptors that can identify and make a reaction to stimulates *via* the innate immune and inflammatory response mechanisms. TLR4 was the first detected protein of the human toll homolog family that could be triggered by lipopolysaccharides (LPS) and provoked proinflammatory cytokines secretion (133) while found to be expressed in both cells and tissues of lung cancer (134, 135). Programmed-death ligand 1 (PD-L1) is recognized as an essential component in the process of immune escape in NSCLC as PD1/PD-L1 pathway has been proved as a vital inhibitory mechanism in lung cancer cells. Triggering of the pathway causes exhaustion of effector T cell and immune escape (136, 137). Studies also suggest that the expression of TLR4 and PD-L1 can indicate the prognosis of NSCLC, while TLR4 may increase expression of PD-L1 *via* the ERK signaling pathway (138). Systemic immune-inflammation index (SII) was found related to poor survival of NSCLC, the prognostic role of which was presented among NSCLC patients with solid nodules, adenocarcinoma, and stage I disease (139).

It has been demonstrated that epigenetic modifications could be triggered by environmental stimuli and it played a vital role in the transcription of inflammatory gene (140). Theoretically, in short-lived inflammatory responses, consecutive epigenetic modifications of pro- and anti-inflammatory molecules can occur. It gave rise to acute inflammation at initial stages of the response followed by epigenetic changes that evoke anti-inflammatory action to end the inflammation (141). In addition, comprehensive epigenome-wide association studies (EWAS) *via* large-scale bioinformatics analysis declared that several epigenetic marks were associated with different circulatory inflammation markers (142). Among the fundamental genes, NF-κB monitored pro- and anti-inflammatory cytokine expression. During initial inflammatory responses, epigenetic landscape changes could lead to the activation of p65, which then gathered to form an activating complex in inflammatory cells. At later stages, epigenetic re-programming of p65 promoter ensued corresponding with chromatin remodeling of the proinflammatory Tnfsf1α and Il1β genes (143, 144). Subsequently, histone methyltransferases, DNMTs, and other chromatin modifiers produced a repressor complex to diminish the inflammatory response (145).

Hence, abnormal epigenetic alterations may aggravate inflammatory responses and affect the risk of chronic inflammatory disease (146). A better knowledge of how inflammation affects epigenetic factors in NSCLC may provide us novel therapeutic strategies. In fact, molecular mechanisms beneath inflammation-associated tumorigenesis have been a vital research area of cancer (147). Researchers revealed genomic aberrations that can help clarify the cause and progression of a variety of cancers and might help to improve current immunotherapy.

## Tumor Microenvironment

It has been described that tumor grows in a complicated and dynamic microenvironment, containing stromal cells, innate cells, endothelial cells, and lymphocytes existing nearby or within the malignant tumors, interacting with each other and the malignant cells. Evidence showed that the complex TME could mediate tumor growth, invasion and metastasis (148). Recently, the investigation of NSCLC metastasis has developed to involve non-cancer cell elements of tumors, the extra-cellular matrix (ECM) components, and stromal cellular compartment comprising the tumor-microenvironment (149). The existence of immune cells, especially the cytotoxic CD8 T cells, within the TME, has an effect on prognosis of tumor. Other immune cells are generally related to tumor progression and poor outcomes involving M2 polarized macrophages, neutrophils, and FOXP3 positive regulatory T cells (150–152). Dieu-Nosjean et al. (153) found that in lung cancers, tertiary lymphoid structures (TLS) involving mature dendritic cells (DC), proliferating B cells, T cells, and follicular DC, presented in the tumor stroma of early-stage NSCLC. In addition, Ning et al. (154) found that histone deacetylase 9 (HDAC9) insufficiency was beneficial for tumor progress *via* reducing infiltration of CD8^+^ DCs in the TME. Compared with wild-type mice, the tumor-infiltrating DCs of Hdac9^−/−^ mice presented decreased cross-presentation of tumor antigens and cross-priming of CD8^+^ T cells. Besides, HDAC9 expression was positively associated with CD8^+^ cell counts significantly in the stroma samples of human lung cancer while lack of HDAC9 reduced inflammation and advocated progress of tumor by reducing CD8^+^ DC infiltration in the TME. Recently, the attention of research on NSCLC drug targets detection has turned from analyzing autonomous functions of cancer cells involving the TME (148). One of the main reasons is that cancer cells involved in both primary tumors and metastatic sites are implicated in various interplays among autocrine and paracrine signaling factors, stromal cells, and ECM-components. Primary tumors must engage blood vessels to promote tumor cell dissemination (angiogenesis), which contains interactions of tumor-cell-endothelial cell and the recruitment of blood vessels by growth factors, namely, CXCL12, FGF, and VEGF-A. Besides, for lung squamous cell carcinoma (LUSC) and LUAD are two major subtypes of NSCLC, Seo et al. analyzed 101 LUSCs and 87 LUADs tumor samples and detected that several micro-environmental factors differentially stimulate immune subtypes of LUAD or LUSC and the expression of the immune checkpoint. Particularly, TAMs are vital immune cells having important impacts on inflammation and TMEs of LUSCs, while regulatory B cells presented as having immunosuppressive and tumorigenic roles in LUADs. The cytolytic activity of CD8^+^ T cell can be reduced by the profusion of macrophages and B cells among immune-competent subtypes. Hence, detecting immune subtypes in NSCLC and their influence on TME may improve clinical evaluating tools for LUADs and LUSCs patients, which also benefit the efficacy of immunotherapy for NSCLC (155). Furthermore, environmental nutrient amounts affect the metabolism of cancer cells, leading to environment-dependent gene essentiality (156, 157). Within TMEs, epigenetic alterations can act a significant role by influencing inflammatory activities. For example, DNA demethylation increases the expression of tumor suppressor gene, thus regaining the tumor prevention while decreasing pro-inflammatory cytokine expression *via* the mediation of ncRNAs or histone modifications, eventually reducing inflammation infiltration in TME (147).

Since lung cancer is one highly heterogeneous disease, cancer cells and cells within the TME together decide the progression of disease and escape from or respond to treatments. To deeply characterize the lung tumor TME, various single-cell resolutions were used for better exploration. Single-cell approach could provide clear visions into the entire tumor ecosystem, namely, mechanisms of intratumoral and intertumoral heterogeneity and cell–cell interactions *via* ligand-receptor signaling (158). Isolated infiltrating T cells in NSCLC were categorized by their functional states and dynamics. A subset of regulatory T cells (Tregs) was reported to correspond to the poor prognosis in LUAD (159). Tumor-infiltrating myeloid cells (TIMs) containing dendritic, macrophage, monocyte, and granulocyte cell lineages, were classified as at least 25 different states by single-cell RNA-sequencing (scRNA-seq) (160). For mapping the cell type-specific transcriptome landscape of cancer cells and their TME in advanced NSCLC, Wu et al. examined 42 tissue biopsy samples from stage III/IV NSCLC patients by scRNA-seq and showed the large scale, single cell resolution profiles of advanced NSCLCs. They found that tumors from different patients present large heterogeneity in chromosomal structure, cellular composition, developmental trajectory, intercellular signaling network, and phenotype dominance (161). Better understanding of factors within TME and their function in NSCLC could help to shed light on the mechanism of NSCLC development and resistance, and provide more feasible options for NSCLC therapy.

## Epigenetics in the Innovative Diagnostic and Therapeutic Strategies

### Innovative Diagnosis

Over the last decade, tissue and/or blood biomarker testing has become popular in treatment decisions for advanced NSCLC. Patients were classified into different biomarker-defined subgroups for targeted and effective therapy, with evidence indicating superior clinical efficacy and less adverse effects compared with traditional cytotoxic chemotherapy (162). With the heterogeneity character of NSCLC and development of epigenetics, novel feasible epi-biomarkers could help to guide more precise and individual therapeutic regimen. The detection of hypermethylation of genes such as CDKN2A, HOXA1, CDX2, and OPCML independently characterized LUAD from healthy samples with 67–86% sensitivity and 74–82% specificity while remarkable DNA methylation was found even in stage I tumor samples (163). Moreover, it was also reported that the methylation degree of SFRP1, p16, KLK10, and DAPK in circulating blood of NSCLC has a great difference compared to normal lung donors and benign lung lesions (164, 165). Hence, they were recognized as potential innovative markers that benefit the early-stage lung cancer diagnosis (166). The gene encoding MLL3 histone methyltransferase, KMT2C, promoter methylation of which in plasma cell‐free DNA (cfDNA) was found to can indicate unfortunate results in NSCLC and provide further assessment as a circulating epigenetic biomarker (167). Sun et al. (168) reported that epigenetic silencing of lncRNA SPRY4 intronic transcript 1 (SPRY4-IT1) occurs in NSCLC cells by direct transcriptional suppression regulated by the Polycomb group protein EZH2 and patients lacking expression of SPRY4-IT1 had poor overall survival, which means that SPRY4-IT1 can be recognized as a useful biomarker for NSCLC prognosis. Tripartite motif containing 27 (TRIM27) is high-expressed in NSCLC. The relevance between CpG methylation of TRIM27 and overall survival of NSCLC patients was estimated by assessing DNA methylation of LUAD and LUSC samples of 613 early-stage NSCLC patients that cg05293407_TRIM27_ methylation can be a possible LUSC prognosis indicator, and smoking levels may influence its predictive significance among different kinds of NSCLC (169). Besides, the mRNA levels of seven epigenetic regulating genes, EZH2, PCNA, RAD54L, SUV39H2, TTF2, UHRF1, and WHSC1, were notably different between NSCLC patients and normal lung tissues (170). The most enriched GO terms were rhythmic process and DNA repair. However, lysine degradation pathway was the most enriched KEGG pathway, which was detected by functional enrichment analysis of the seven genes. These findings validate that EZH2, RAD54L, UHRF1, and WHSC1 are prospective predictive biomarkers to characterize NSCLC patients of high or low risk. The significance of correct chromatin composition is highlighted by the signature of ATP-dependent chromatin remodeling complexes in disease. These are multisubunit complexes that can move and transfer nucleosomes, thus mediating transcription. Various elements of the highly conserved SWI–SNF complex have been linked to cancer, for example, the ATPase subunits BRM and BRG1 are mutated in several cancer cell lines and primary tumors, which is correlated to a poor prognosis of NSCLC patients (171).

On the other hand, HDAC as epigenetic regulators have been applied clinically for treating hematopoietic malignancies. Recently, HDACi was recognized as a component mediating the immune system and its expression was described to foresee the development of NSCLC patients who received treatment of immune checkpoint inhibitors (ICIs). Additionally, HDACi combined with PD-1 inhibitor showed efficacy in inhibiting tumor growth and provide better TME for cytotoxic T cells in TC-1 mouse model (172). N6-methyladenosine (m^6^A) is one of the most frequent epigenetic alterations in eukaryotic RNA, which is a reversible process and plays a critical role in various diseases including cancers. The m^6^A modification of RNA, mediated by demethylases, methyltransferases, and m^6^A-binding molecules, influences the progress of NSCLC by affecting the target RNA splicing, translation, decay, and nuclear export. It has been recommended that the influence of m^6^A modification on the prognosis of NSCLC patients is a double-edged sword, indicating m^6^A modification has both promotive and inhibitory effects on progress of NSCLC (173). CircRNAs are a class of conserved single-stranded RNA molecules derived from intronic or exonic sequences by back-splicing of precursor mRNA, and have been described to have effects on microRNA sponges, regulators of gene splicing and transcription, RNA-binding protein sponges, and protein/peptide translators (174). Increasing evidence shows that circRNAs can function as predictive biomarkers for NSCLC. For example, Wang et al. (106) verified that circPTK2 (hsa_circ_0008305) sponged miR-429, thus promoting cell invasion of NSCLC. Circular RNA circFGFR1 was observed to have high levels among NSCLC patients, which was associated with poor NSCLC prognosis (175). LncRNAs were linked to several cellular activities and alteration of lncRNAs expression may accelerate development of tumor, involving NSCLC (176). Evidence indicated that Hox transcript antisense RNA (HOTAIR) could directly modulate cancer progression and can be used as a potential prognostic biomarker for NSCLC (177). Among patients with genotype of GG polymorphism, the expression level of colon cancer-associated transcript 2 (CCAT2) transcripts was enhanced, demonstrating that CCAT2 can be a new biomarker for metastasis of cancer (178) and possibly contributes to cancerogenesis and metastasis as one of oncogenic lncRNAs (179). There are other lncRNAs that have been characterized as promising biomarkers of NSCLC such as LCAL (180), AFAP1-AS1 (181), and linc00673 (103). It was indicated that immunity condition determined with markers of DNA methylation was linked to lung cancer prior to the cancer diagnosis. Thus, a better insight of immunity-related methylation biomarkers in lung cancer progress could offer vision to fast and precise diagnosis and treatments (182). Dysregulated inflammation was known as one of the hallmarks of cancer and was associated with tumor origination, development, and metastasis (183–185). Interleukin-1 beta (IL-1β), a proinflammatory cytokine, associates with NSCLC progress and was found a higher level in serum of NSCLC patients than in healthy donors while increased IL-1β in these patients correlated with poor survival (186).

### Therapy

Innovative therapeutic patterns are central for improving the existing immunotherapy in NSCLC. Studies have shown that significant functions of epigenetic processed in mediating immune cell function and regulating antitumor immunity. Interactions between them have promoted consolidation of epigenetic therapy and immunotherapy (**Figure 1**). An appealing method to overcome the restrictions of immunotherapy alone is in demand (187). Several epigenetic therapies for NSCLC were carried out in clinical trials (**Table 1**). Detecting the molecular characteristics of NSCLC subtypes, such as genetics and epigenetic variation, was critical for choosing the proper therapy for combination (13) while chemotherapy resistance was also found to be associated with epigenetic changes (188). Besides, DNA methylation is a double-sided procedure, unlike the changes of genetic information involving gene mutations or deletions. Hence, in theory, demethylation treatment on patients with lung cancer or precancerous lesions might repair the function of certain tumor suppressor genes, thus reaching the goal of treating or guarding against lung cancer (166). The hypermethylation of p16, as a tumor suppressor, was demonstrated to make contribution to the clinical treatment and may be used as a biomarker for early diagnosis of NSCLC (189, 190). Research explored DNA methylation markers as a modality for the early diagnosis of lung cancer and were helpful in the therapy process (191). Additionally, as has been discussed before, quite a few HDACi were studied for their anti-tumor efficacy (192). During radiotherapy, radio-protective autophagy and histone H4 lysine 20 trimethylation (H4K20me3) were found to be upregulated after irradiation, regulation of which axis may be a new approach to improve radiotherapy for NSCLC (193). Since bromodomain functions as an epigenetic reader element for acetylated lysine on histone or non-histone molecules, the bromodomain and extra-terminal (BET) proteins that residing on vigorous promoter and enhancer regions (194), were functionally related to transcriptional co-activators that positively control RNA Pol II-dependent transcription. Plenty of BET inhibitors (BETi) displayed strong antitumor effects among several cancer kinds (195–197). Moreover, the fast progress of target therapies primarily in lung cancer involved certain oncogenic proteins, especially ALK and EGFR mutations (198–200). For EGFR mutations, generations of TKIs were developed and were used in the treatment of lung cancer (201). Increasing evidence demonstrated that BETi could synergize with TKIs to improve antitumor efficacy in a range of cancer types (202–204).

**Table 1:** Summary of epigenetic approaches for NSCLC in clinic.

Tumor type	Drug	Study size	Developer	Highest trial stage
NSCLC	Azacitidine	120	Sidney Kimmel Comprehensive Cancer Center at Johns Hopkins	Phase 2
	Entinostat			
	Nivolumab			
Carcinoma, NSCLC, Lung Cancer, Esophageal, Malignant Pleural Mesotheliomas	Decitabine (DAC)	85	National Cancer Institute (NCI)	Phase 2
	Tetrahydrouridine (THU)			
	Pembrolizumab			
NSCLC	Nivolumab oral decitabine	13	Case Comprehensive Cancer Center	Phase 2
	Tetrahydrouridine			
Carcinoma, NSCLC	nab-paclitaxel IV	240	Celgene	Phase 2
	CC-486			
	Duravalumab			
Lung Cancer	Pembrolizumab	28	Memorial Sloan Kettering Cancer Center	Phase 1
	Guadecitabine			
	Mocetinostat			
Small Cell Carcinoma, Carcinoma, NSCLC, Neuroendocrine Tumors, Ovarian Epithelial Cancer	RRx-001	213	EpicentRx, Inc.	Phase 2
	Cisplatin			
	Etoposide			
	Carboplatin			
	Irinotecan			
	Vinorelbine			
	Doxil			
	Gemcitabine			
	Taxane			
	Paclitaxel			
	Nab-Paclitaxel			
	Pemetrexed			
Carcinoma, NSCLC	CC-486	100	Celgene	Phase 2
	Pembrolizumab			
	Placebo			

ICIs, especially inhibitors of the PD-1 immune-checkpoint axis, have modified the treatment of NSCLC during the last decade (205). Though melanoma was the most responsible solid tumor toward immunotherapy (185), encouraging outcomes were accomplished in advanced NSCLC that was one of the most lethal cancers (185, 206, 207). FDA have approved trials for melanoma and NSCLC with promising results (208). Studies of an early phase I/II clinical trial of the combined use of epigenetic therapy and the HDACi entinostat and the DNMTi azacitidine (9) have promoted to make the notion of combination therapy of epigenetic drugs and ICI. One research involved a small quantity of advanced NSCLC patients taking low-dose epigenetic therapy enrolled a trial of immune checkpoint therapy. About 20% of the patients responded well, with no progression for 24 weeks and the majority reaching high-grade Response Evaluation Criteria in Solid Tumors (RECIST) criteria responses (206, 209) which was an overwhelming achievement for immunotherapy in NSCLC (210). Preclinical data also suggested that agents such as HDACi could present an exclusive capacity of transforming TMEs into an immunotherapy-profitable condition (211). Based on current data from early-stage clinical trials in NSCLC, such combination may improve tumor reactions to immunotherapy or recover responses to immunotherapy for those who suffer from treatment resistance.

Inflammation is linked to the initiation and progress of cancer. Inflammation has recently been identified as one of the promoting signatures of cancer (185). Around 25% of cancers were related to chronic inflammation by some means (212), which was linked to lung cancer as a result of constant exposure to factors in tobacco smoke (213, 214). Besides, the cells in charge of cancer-related inflammation were inherently steady, indicating that they are not suffered from the common occurrence of drug resistance. Thus, targeting inflammation can be a promising approach for both cancer prevention and treatment (107). Cisplatin-based chemotherapy remains one of the standard cares for NSCLC patients. Recrudesce after chemotherapy-caused dormancy influence the overall survival. The change of cancer cells undergoing chemotherapy tension was measured by transcription factors (TFs) *via* binding sites primarily buried deep within inaccessible chromatin. Several key TFs regulated gene expressions during the process of dormancy and reactivation of lung cancer cells through modifying promoter accessibility of target genes (215). As one of the most common oncogenic drivers in NSCLC, KRAS mutations account for about 25% of LUAC (216, 217). It was reported that co-appearing genomic variations in the STK11/LKB1 (KL) and TP53 (KP) tumor suppressor genes modify subgroups of KRAS-mutant LUAC with distinct biology, immune profiles, and therapeutic vulnerabilities (218). The effects of STK11/LKB1 alterations on clinical efficacy of PD-1/PD-L1 inhibitors expanded to PD-L1-positive NSCLC. In Kras-mutant murine LUAC models, Stk11/Lkb1 loss enhanced PD-1/PD-L1 inhibitor resistance, suggesting alterations of STK11/LKB1 as a key driver of PD-1 blockade resistance in KRAS-mutant LUAC (219).

Recently, gene therapy using clustered regularly interspaced short palindromic repeats (CRISPR)-Cas9 technique has achieved popularity among fields of science principally due to its high efficacy, versatility, and cost-effectiveness. The CRISPR-mediated genome editing has been broadly utilized in various cell types and organisms to specifically edit target genes with sgRNA recognizing specific sites (220–222). It has been proved that editing of immune checkpoint genes by CRISPR-Cas9 could enhance the efficacy of T cell therapy. Lu et al. conducted treatment of 12 NSCLC patients with PD-1 gene-edited bulk autologous T cells. The results suggested that both safety and feasibility of gene editing for cell therapy could be observed (223). Svensson et al. (224) investigated the tumorigenesis conditions in the KRAS-LKB1 and KRAS-P53 mouse models and found that knockout of acetyl Co-A carboxylase in NSCLCs was detrimental for tumor growth. To explore epigenetic regulators as novel treating targets for NSCLC, pooled epigenome-wide CRISPR knockout screens were performed both *in vitro* and *in vivo*. The histone chaperone nucleophosmin 1 (Npm1) was detected as a prospective therapeutic target (4). Furthermore, delivering nucleic acid-based therapeutics to cells has become a promising approach to target the genetic cause of various diseases, with the capacity to regulate protein production. Libraries of ionizable lipid nanoparticles (LNPs) were designed to encapsulate mRNA, inhibiting its degradation and facilitate intracellular delivery (225). Besides, a number of siRNA-encapsulated LNPs have been applied in the treatment of intractable diseases, namely, cancer, genetic diseases, and inflammatory neurological disorder (226). Current powerful CAR T cell engineering methods using viral delivery vectors could lead to constant CAR expression and severe adverse reactions. The designed LNPs were utilized to deliver mRNA to primary human T cells to generate functional protein expression, indicating the potent possibility of LNPs to improve mRNA-based CAR T cell engineering methods (227).

Both diagnostic and therapeutic methods are crucial for timely and effective treatment of NSCLC. More exact research on mechanisms beneath epigenetic alteration and inflammation are demanded to provide foundation and recommendation for future treatment of NSCLC. Research can also utilize superior gene editing approaches to enhance treatment efficacy. Epigenetic drugs hold remarkable therapeutic potential to be optimized and utilized for a broad range of NSCLC patients.

## Conclusions

We elucidated epigenetic alteration and inflammation-related carcinogenesis from the aspects of gene modulation and cellular level. The roles that epigenetics and inflammation played in tumor progression are complex and can be used for biomarker detection and therapy for NSCLC. The epigenetic therapy has been described by data from preclinical and clinical studies, providing wide application prospects. With an enhanced understanding of the exact regulating mechanisms that underlie the epigenetics and inflammation influenced factors and target genes of NSCLC, there will be further opportunities to improve the current prognosis for NSCLC and present a new era in the approach for treatment development for NSCLC patients as new anti-tumor drugs or combination with immunotherapy, chemotherapy or other targeted therapy.

## Author Contributions

SY wrote the manuscript. YH and QZ revised the manuscript. All authors listed have made a substantial, direct, and intellectual contribution to the work and approved it for publication.

## Funding

This work was supported by the High-level University Construction Fund of Guangdong Province (06-410-2107217), the National Science Fund for Distinguished Young Scholars of China (82102860), the National Key R&D Program of China (2019YFA0904400), the Shenzhen Science and Technology Project (SGDX2020110309280301), the Guangzhou Science and Technology Project (202102020516), and the Science and Technology Development Fund of Macau (File no. FDCT/0043/2021/A1, FDCT/0002/2021/AKP, and FDCT/0004/2019/AFJ), University of Macau (File no. MYRG2019-00069-FHS).

## Conflict of Interest

The authors declare that the research was conducted in the absence of any commercial or financial relationships that could be construed as a potential conflict of interest.

## Publisher’s Note

All claims expressed in this article are solely those of the authors and do not necessarily represent those of their affiliated organizations, or those of the publisher, the editors and the reviewers. Any product that may be evaluated in this article, or claim that may be made by its manufacturer, is not guaranteed or endorsed by the publisher.
